# Integrated analysis of co‐expression and ceRNA network identifies five lncRNAs as prognostic markers for breast cancer

**DOI:** 10.1111/jcmm.14721

**Published:** 2019-10-15

**Authors:** Yan Yao, Tingting Zhang, Lingyu Qi, Chao Zhou, Junyu Wei, Fubin Feng, Ruijuan Liu, Changgang Sun

**Affiliations:** ^1^ Clinical Medical Colleges Weifang Medical University WeiFang China; ^2^ College of First Clinical Medicine Shandong University of Traditional Chinese Medicine Jinan China; ^3^ Department of Oncology Weifang Traditional Chinese Hospital WeiFang China; ^4^ Department of Oncology Affiliated Hospital of Shandong University of Traditional Chinese Medicine Jinan China; ^5^ Department of Oncology Affiliated Hospital of Weifang Medical University WeiFang China

**Keywords:** breast cancer, ceRNA network, overall survival, The Cancer Genomes Atlas, weighted gene co‐expression network analysis

## Abstract

Long non‐coding RNAs (lncRNAs), which competitively bind miRNAs to regulate target mRNA expression in the competing endogenous RNAs (ceRNAs) network, have attracted increasing attention in breast cancer research. We aim to find more effective therapeutic targets and prognostic markers for breast cancer. LncRNA, mRNA and miRNA expression profiles of breast cancer were downloaded from TCGA database. We screened the top 5000 lncRNAs, top 5000 mRNAs and all miRNAs to perform weighted gene co‐expression network analysis. The correlation between modules and clinical information of breast cancer was identified by Pearson's correlation coefficient. Based on the most relevant modules, we constructed a ceRNA network of breast cancer. Additionally, the standard Kaplan‐Meier univariate curve analysis was adopted to identify the prognosis of lncRNAs. Ultimately, a total of 23 and 5 modules were generated in the lncRNAs/mRNAs and miRNAs co‐expression network, respectively. According to the Green module of lncRNAs/mRNAs and Blue module of miRNAs, our constructed ceRNA network consisted of 52 lncRNAs, 17miRNAs and 79 mRNAs. Through survival analysis, 5 lncRNAs (AL117190.1, COL4A2‐AS1, LINC00184, MEG3 and MIR22HG) were identified as crucial prognostic factors for patients with breast cancer. Taken together, we have identified five novel lncRNAs related to prognosis of breast cancer. Our study has contributed to the deeper understanding of the molecular mechanism of breast cancer and provided novel insights into the use of breast cancer drugs and prognosis.

## 1 INTRODUCTION

Breast cancer, the most frequently diagnosed carcinoma, is the deadliest form of cancer affecting women worldwide.^1^ According to the statistics of the American Cancer Society, more than 268 600 new cases of invasive breast cancer will be diagnosed in 2019, and approximately 41 760 death cases are expected.^2^ Currently, although multiple therapeutic measures are employed, such as chemotherapy, surgery, radiotherapy, endocrine therapy and targeted therapy,^3^ the majority of breast cancers remain incurable. This therefore highlights the urgent need for identifying the molecules that have an inhibitory role or facilitating role in tumours, determining novel relevant treatment targets and further improving the treatment and prognosis of patients with breast cancer. Long non‐coding RNAs (lncRNAs) are a type of RNA that are over 200 nucleotides in length.^4^ Although they do not participate in protein coding, increasing evidence has demonstrated that lncRNAs are involved in the biological processes of cellular proliferation, apoptosis and differentiation.^5^, ^6^ Furthermore, sufficient information has demonstrated that lncRNAs take part in the regulation of tumour progression as well as tumour biological behaviours through interactions with microRNAs (miRNAs) or messenger RNAs (mRNAs).^7^, ^8^, ^9^, ^10^ The competing endogenous RNA (ceRNA) hypothesis proposed by Salmena et al have attracted increasing attention.^11^ This hypothesis has been supported by a large number of experiments. For example, a previous study found that lncRNA‐related ceRNAs play a key role in biological processes of glioblastomas.^12^ Additionally, Ning et.al found that through competing with miR‐331‐3p, the lncRNA HOTAIR, as a ceRNA, could induce and activate human epithelial growth factor receptor 2 (HER2) cell signalling networks.^13^ Nevertheless, little is known about the role of ceRNA in breast cancer.

Weighted gene co‐expression network analysis (WGCNA) is a system biology algorithm commonly used to explore the correlation between gene sets and clinical features via constructing free‐scale gene co‐expression networks.^14^, ^15^, ^16^The advantage of WGCNA is that it can identify and cluster highly correlated genes into the same module. Furthermore, these modules also provide external clinical traits with related modules. However, the majority of studies focused on differentially expressed genes and ignored the high relationship between genes. At present, WGCNA plays a significant role in multiple fields, such as cancer, nervous system and genetic data analysis, which is extremely useful for identifying potential candidate biomarkers or novel treatment targets.^17^, ^18^, ^19^, ^20^

Our study utilized the data related to breast cancer from The Cancer Genomes Atlas (TCGA) database to identify novel lncRNAs. Here, we extracted breast cancer expression profiles of lncRNAs, miRNAs and mRNAs from TCGA database and their corresponding clinical data. Subsequently, we selected lncRNAs, mRNAs and miRNAs whose expression levels were in the top 5000 among the 112 matched specimen pairs. Afterwards, these data were used to construct a co‐expression network to determine the module related to the clinical trait. Based on the data of the module, ceRNA network analysis was performed using the following databases: miRTarBase, miRcode and TargetScan. Additionally, we also performed survival analysis to screen lncRNAs most relevant to prognosis. Findings from our study contribute to further understanding of the molecular mechanisms, biological processes and treatment targets of lncRNAs in the field of breast cancer research.

## 2 MATERIALS AND METHODS

### 2.1 Data acquisition and pre‐processing

As the largest cancer gene information database at present, TCGA database provides not only a variety of cancer types but also multi‐omics data, including gene transcript, miRNA expression data and DNA methylation. Additionally, it also has the advantage of containing abundant and standardized clinical data, as well as large sample sizes for each cancer type. All data sets were downloaded from TCGA (https://portal.gdc.cancer.gov/, accessed in 18 December, 2018）database through the package TCGAbiolinks in R software (version 3.5.1), including lncRNA, mRNA and miRNA expression profiles of breast cancer specimens and the corresponding clinical follow‐up data. Notably, we performed data analysis on the basis of ‘Level‐3’ read counts. The data used in this study met the following criteria: (a) there were pairs of cancerous and the corresponding normal tissue in the datasets; and (b) they had specific follow‐up times. Based on the TCGA ethics committee, we obtained these relevant data that are open access and public; thus, ulterior approval was not required.

### 2.2 Construction of weighted gene co‐expression network

The expression profile of these genes, lncRNAs and miRNAs were applied to construct a gene co‐expression network by using the package WGCNA implemented in R software.^14^ We performed the same analysis as described previously.^14^ The construction process of genes and lncRNAs co‐expression network is similar to that of miRNAs co‐expression network with the exception of some parameters. For example, we constructed a genes and lncRNAs co‐expression network.

This procedure included the following key steps: firstly, in order to eliminate the interference caused by the length of gene and the depth of sequencing, the method of Fragments Per Kilobase Million (FPKM) was applied to standardize the data of ‘Level‐3’ read counts. The formula of FPKM is as follows:

$$\text{FPKM}=\frac{\text{total}\;\text{gene}\;\text{Fragements}}{\text{mapped}\;\text{Reads(Millions)}\times \text{gene}\;\text{Length(KB)}}$$

In this study, FPKM values were >1 in more than 50% of specimen pairs. Then, through drawing cluster trees, outlier samples were removed in order to make the results more credible. Secondly, we calculated Pearson's correlation coefficient (PCC) cor (*i*,*j*) for each pair of mRNAs and lncRNAs. The construction of similar expression matrix is as follows:

$$a_{\mathrm{ij}}{=(0.5\times \left(1+\text{cor}\left(\;i,j\right)\right)}^{\beta }$$

In which, *a_ij_* refers to the contributions between genes *i* and *j*. Subsequently, one needs to introduce a power of *β* value so that it could transform the similarity matrix into an adjacency matrix. For mRNAs and lncRNAs, the power of *β* value is 10; however, for miRNAs, the power of *β* value is 6. On this basis, we constructed a scale‐free network and topological overlap matrix (TOM). After that, we also carried out the corresponding dissimilarity of TOM (dissTOM), from which hierarchical clustering tree of genes (dendrogram) by function hclust was produced by hierarchical clustering for module detection. The Dynamic Tree Cut method was applied to generate modules with the following major parameters to avoid generation of too many modules: deepSplit of 2 and minModuleSize of 40 (For miRNAs, the minModuleSize was set as 20). The height cut‐off was set as 0.25, modules were merged together if their similarity was >0.75. The cut‐off was set as 0.20 for miRNAs. Ultimately, these mRNAs and lncRNAs containing co‐expression modules are considered to be highly interconnected.

### 2.3 Relationship between clinical information and modules

The correlation between modules and clinical information (ie normal or tumour) of breast cancer was identified by PCC. Above all, module eigengenes (MEs) referred to the first principal component of all gene expression levels in the module, and therefore, it was reasonable to consider that MEs represented all genes within a specific module. According to Pearson's correlation tests, we further identified the association between MEs and external clinical information including sample status. If *P*‐value was < .05, it was considered to be a significant correlation.

### 2.4 CeRNA network construction and analysis

According to the results of WGCNA, we selected all mRNAs, lncRNAs and miRNAs in the most relevant module to construct a ceRNA network. Briefly, the associated ceRNA network in breast cancer was constructed following three stages. (a) Prediction of lncRNA‐miRNA: in order to make lncRNAs and miRNAs map into the interactions successfully, we used the online miRcode (http://www.mircode.org/) database.^21^ MiRcode contains ‘whole transcriptome’ human miRNA target predictions on the basis of the comprehensive GENCODE gene annotation, consisting of more than 10 000 lncRNAs. (b) Prediction of miRNA‐mRNA: firstly, the names of DEmiRNAs were transformed into human mature miRNA names using starBase v2.0 (http://starbase.sysu.edu.cn) online tool. Secondly, three online databases, TargetScan (http://www.targetscan.org/),^22^ miRDB (http://www.mirdb.org/miRDB/)^23^ and miRTarBase (http://mirtarbase.mbc.nctu.edu.tw),^24^ were used simultaneously for target mRNA prediction. Additionally, we also used the package Venny in Rstudio software to obtain the overlaps for ensuring more credible results. (c) Construction of lncRNA‐miRNA‐mRNA ceRNA network: Cytoscape 3.7.0 software was used to construct and visualize the ceRNA network based upon lncRNA‐miRNA and miRNA‐mRNA pairs.^25^ Furthermore, for all lncRNAs in the ceRNA network, we used the edgeR package in R software for differential expression. The cut‐off threshold was |log_2_FC > 1| and adjusted *P*‐value < .05.

### 2.5 Gene ontology and pathway enrichment analysis

The Database for Annotation, Visualization and Integrated Discovery (DAVID [version 6.8]; https://david.ncifcrc.gov/)^26^ has a comprehensive set of functional annotation tools for investigators to more deeply understand the biological meaning behind a large list of genes. DAVID online tool was used to perform Gene Ontology (GO) and Kyoto Encyclopedia of Genes and Genomes (KEGG) pathway analyses for overlapping targeted genes. GO consists of the following three parts: biological process (BP), molecular function (MF) and cellular component (CC). Additionally, all significant GO or KEGG terms or genes, with significance of *P* < .05, should be comprised of at least two mRNAs. The results of enrichment analysis were visualized using the package GOplot in R software.

### 2.6 Survival analysis

The standard Kaplan‐Meier univariate curve analysis was adopted to identify the prognosis of lncRNAs. In addition, we combined analysis with the clinical information of those samples in TCGA database and drew survival curves of all lncRNAs in the ceRNA network. Meanwhile, for overall survival, the high‐expression and low‐expression cohorts were split for the log‐rank test through the package survival in R software. When the *P*‐value of lncRNAs was <.05, it was considered to be statistically significant, which meant that the lncRNA had potential prognosis.

## 3 RESULTS

### 3.1 Pre‐processing of the data sets

Expression data of lncRNAs and mRNAs were collected from 1222 specimens, which were composed of 1109 tumorous and 113 normal samples, and included 112 pairs of matched tumorous and normal samples. Eventually, we obtained a total of 19 983 mRNAs and 14 384 lncRNAs. After obtaining the expression data, we standardized these data sets by using the method of quantile normalization. According to the sum of expression quantity of each gene or lncRNA, we ranked them from largest to smallest and only selected the top 5000 genes and lncRNAs for further analyses. For miRNAs, if their expression levels were higher than 1 in more than 50% of the 102 matched pairs, then they were selected for WGCNA. Next, 665 miRNAs in total were chosen to perform for subsequent analyses.

### 3.2 Construction of weighted co‐expression network and identification of key modules

**Figure 1:**
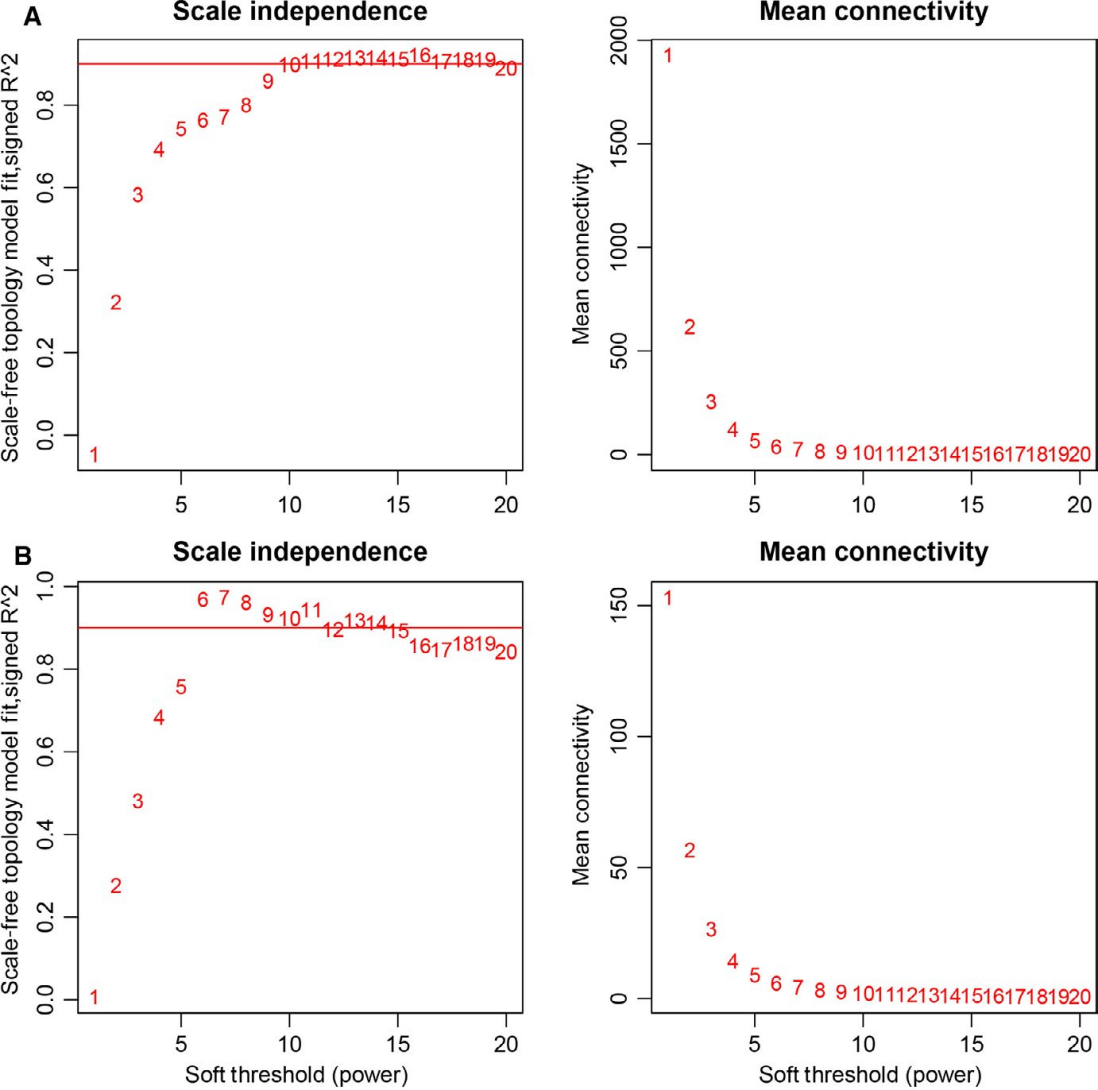
A, Determination of soft‐thresholding power in the lncRNAs/mRNAs WGCNA. B, Determination of soft‐thresholding power in the miRNAs WGCNA. Left: Analysis of the scale‐free fit index for various soft‐thresholding powers (β). Right: Analysis of the mean connectivity for various soft‐thresholding powers

**Figure 2:**
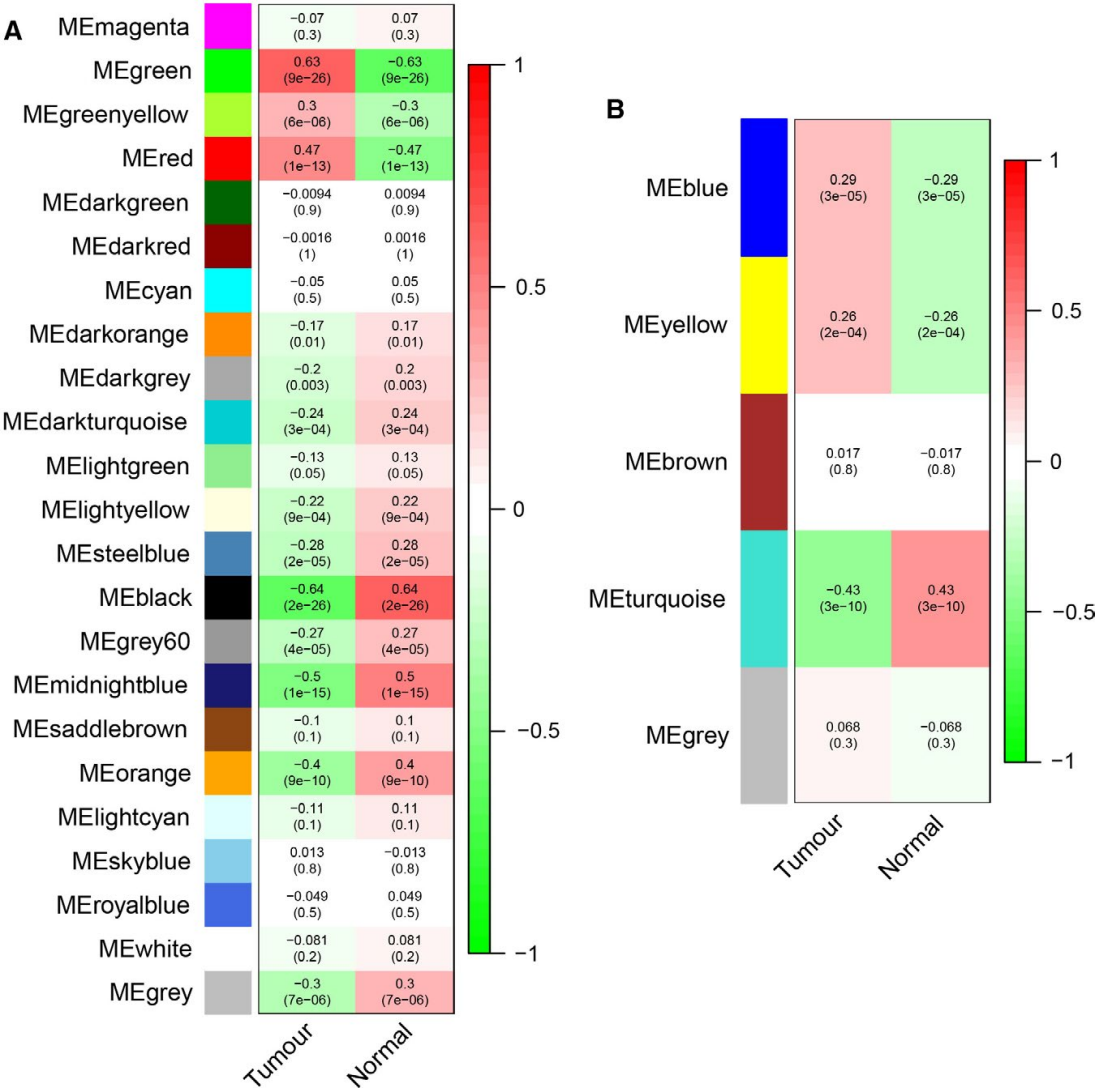
A, Clustering dendrogram of lncRNAs and mRNAs. B, Clustering dendrogram of miRNAs. Note: The hierarchical clustering tree was produced by hierarchical clustering based on dissTOM of genes. In the coloured rows below the dendrogram, the two coloured rows represent the original modules and merged modules

**Figure 3:**
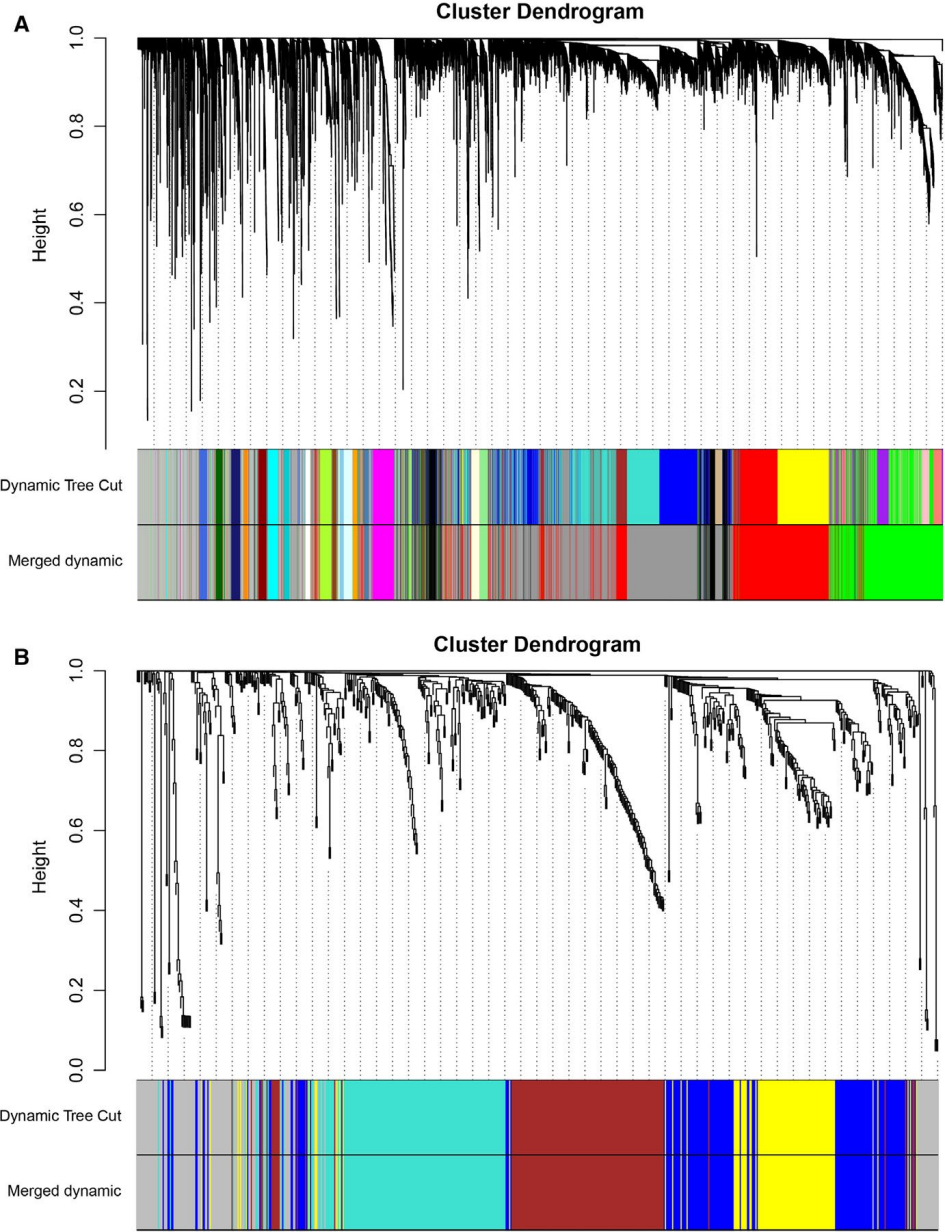
A, Module‐trait associations of lncRNAs and mRNAs were evaluated by correlations between MEs and clinical traits. B, Module‐trait associations of miRNAs were evaluated by correlations between MEs and clinical traits. Note: Each row corresponds to a module eigengene, and each column corresponds to a trait. Each cell contains the corresponding correlation (first line) and *P*‐value (second line). The table is colour‐coded by correlation according to the colour legend. *P*‐values < .05 represent statistical significance

The expression profiles of 5000 lncRNAs, 5000 mRNAs and 665 miRNAs were obtained for constructing the co‐expression network via the package WGCNA in R software. In our study, the most important step is how to select the soft‐threshold power. To determine the relative equilibrium between scale independence and mean connectivity, we analysed the network topology with soft‐threshold power from 1 to 20. To this end, we eventually confirmed *β* values of 10 and 6 in lncRNAs/mRNAs and miRNAs co‐expression network analysis, respectively (Figure 1A,B). Next, the method of dynamic tree cutting was employed to produce co‐expression modules. Afterwards, the minimum number of mRNAs and lncRNAs in each module was set as 30. Additionally, the parameter of MEDissThres was set as 0.25 to merge closely associated modules into larger ones. In the miRNA co‐expression network, the minimum number of each module was set as 20 and the parameter of MEDissThres was set as 0.20. Ultimately, a total of 23 and 5 modules were generated in the lncRNAs/mRNAs and miRNAs co‐expression network, respectively (Figure 2A,B). It is worth noting that we calculated and plotted the relation of each module with their corresponding clinical traits. From Figure 3A, we could conclude that the Green module revealed the strongest positive correlation (module‐trait weighted correlation = .63) with tumour samples related to the lncRNAs and mRNAs co‐expression network. Meanwhile, as shown in Figure 3B, the Blue module has positively correlated (module‐trait weighted correlation = .29) with tumour samples related to the miRNAs co‐expression network.

### 3.3 ceRNA network in breast cancer

**Figure 4:**
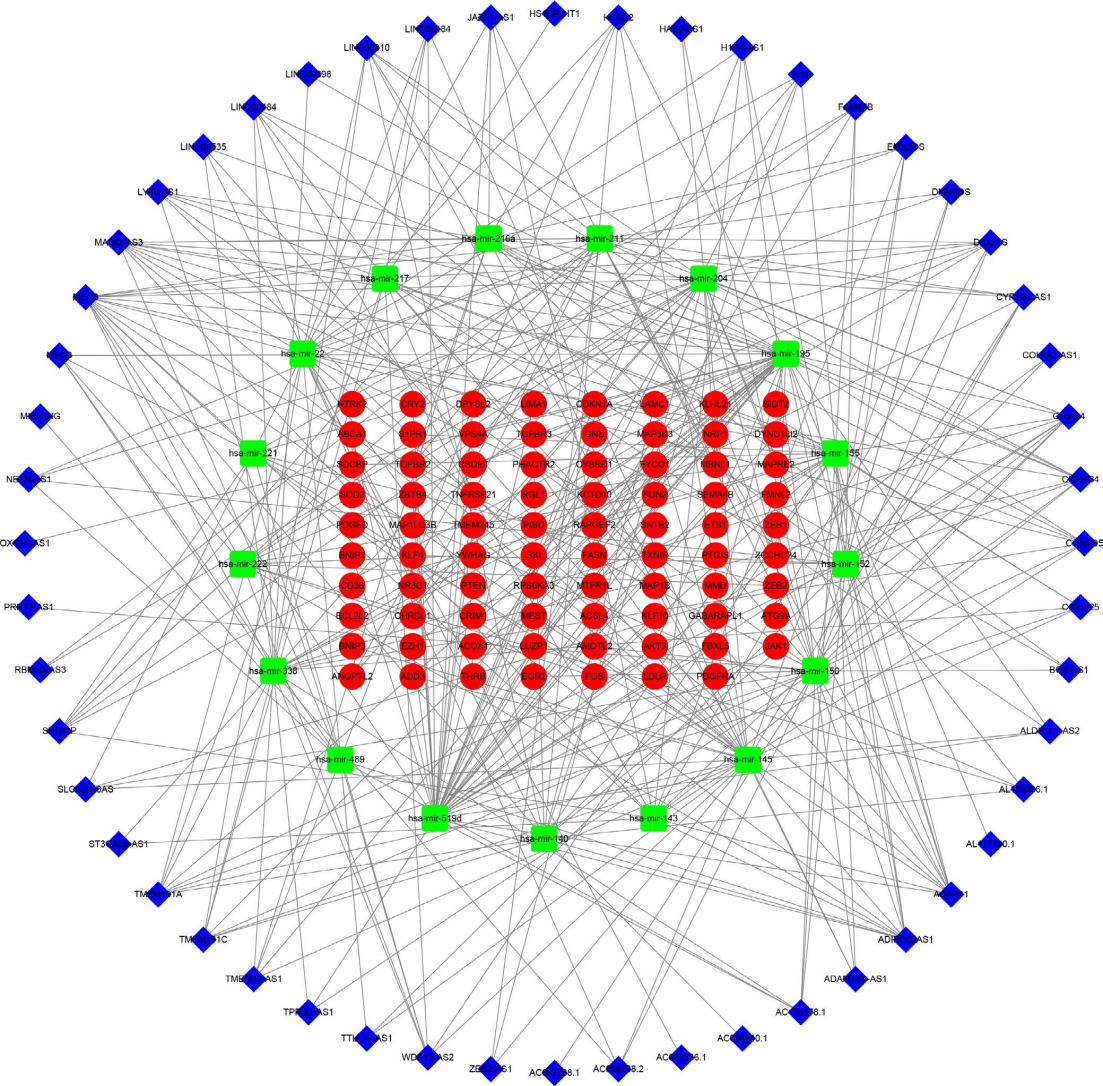
Green module of lncRNA and mRNA and Blue module of miRNA ceRNA network. Notes: Blue diamond denotes lncRNA, green square represents miRNA, and red round rectangle represents mRNA

Firstly, based on the miRcode online database that matches potential miRNAs with lncRNAs, a total of 257 lncRNA‐miRNA pairs contained 52 lncRNAs (4 up‐regulated and 44 down‐regulated) and 17 miRNAs. Next, with regard to the target gene predictions of the 17 miRNAs, we used the following online tools: TargetScan, miRDB and miRTarBase, which consisted of 1162 miRNA‐mRNA pairs, including 966 target genes. Subsequently, we matched the predicted target gene with the mRNAs in the Green module. Finally, we obtained 79 target mRNAs overall. On the basis of the results above and with the inclusion of 52 lncRNAs, 17miRNAs and 79 mRNAs, we performed and visualized the lncRNA‐miRNA‐mRNA network using Cytoscape version 3.7.0 software (Figure 4).

### 3.4 Enrichment analysis of Gene Ontology and KEGG pathways

**Figure 5:**
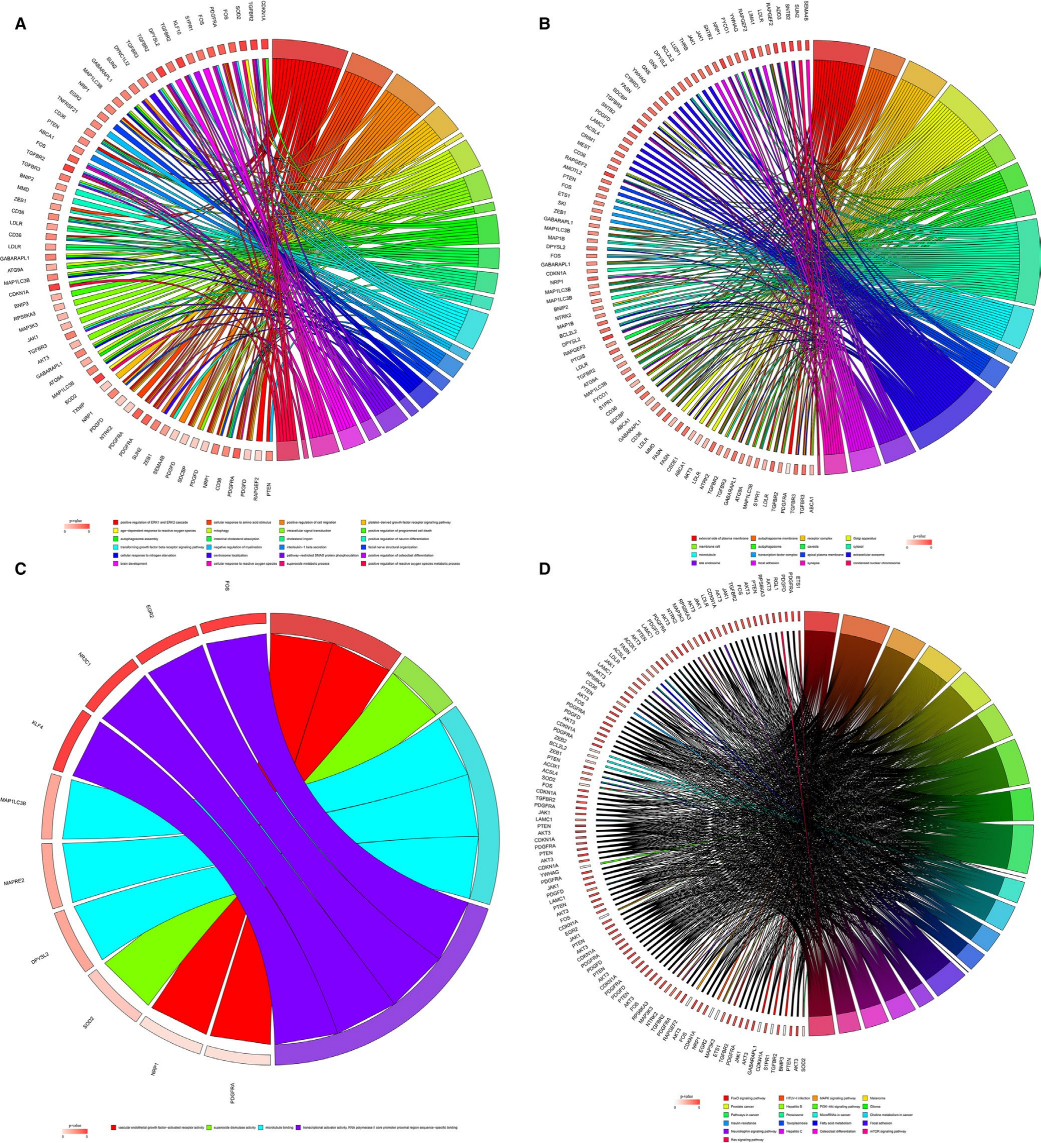
Enrichment analyses of overlapped genes. A, Biological process; B, cellular component; C, molecular function; D, Kyoto Encyclopedia of Genes and Genomes (KEGG) pathways. Note: Chord plot displays the relationship between genes and terms

To further clarify the potential biological functions of mRNAs in breast cancer, DAVID online tool was used to perform functional enrichment analysis. From the 79 target mRNAs, a total of 30 GO terms were enriched. In particular, these mRNAs are primarily associated with intracellular signal transduction, positive regulation of programmed cell death, transforming growth factor beta receptor signalling pathway and autophagosome. In addition, from the 79 target mRNAs, a total of 21 KEGG pathways were enriched. Among these enriched pathways, many are tumour‐related, such as MAPK signalling pathway, PI3K‐Akt signalling pathway, pathways in cancer, microRNAs in cancer, mTOR signalling pathway and Ras signalling pathway. The visualization results are shown in Figure 5.

### 3.5 Survival analysis

**Figure 6:**
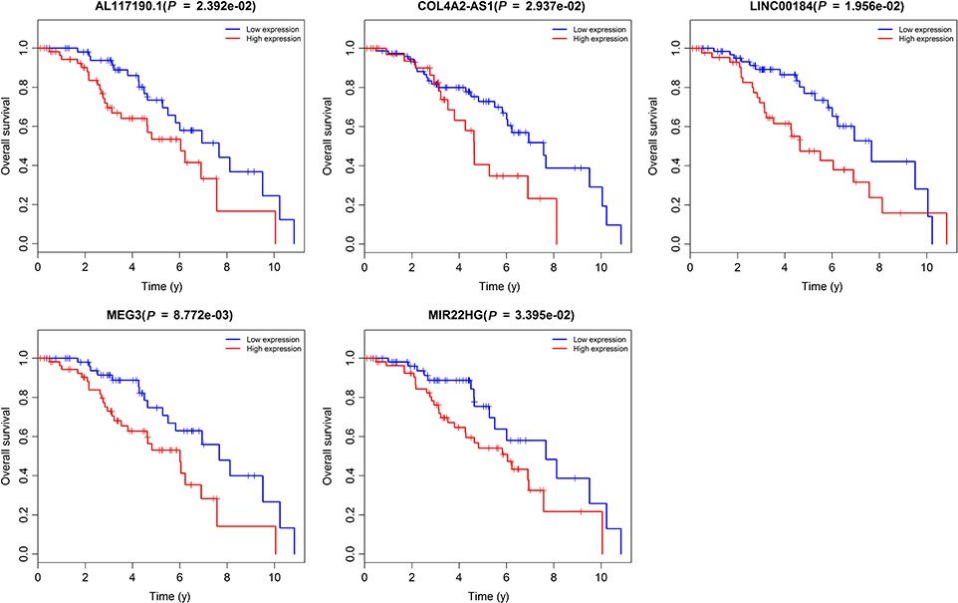
Kaplan‐Meier survival curves of five lncRNAs in the ceRNA network

Kaplan‐Meier and log‐rank test based on the package survival in R software were used to identify the correlation between all lncRNAs in the ceRNA network and overall survival using *P* < .05 as a cut‐off threshold. Of the 52 lncRNAs, Kaplan‐Meier revealed that 5 lncRNAs (AL117190.1, COL4A2‐AS1, LINC00184, MEG3 and MIR22HG) were identified as crucial prognostic factors. Notably, low expression of 5 lncRNAs has a better prognosis in patients with breast cancer (Figure 6). When the expression of 5 lncRNAs is high, the survival time is relatively short in patients with breast cancer; thus, these lncRNAs could be related to poor prognosis.

## 4 DISCUSSION

Women with breast cancer have a higher rate of distant metastasis and a poorer prognosis than those with other malignant tumours. The current poor overall survival among patients with breast cancer could be due to the lack of more efficient therapeutic targets and prognostic biomarkers. In the present study, we adopted WGCNA to identify modules, including one lncRNAs/mRNAs module (Green) and one miRNA module (Blue) that were most significantly associated with breast cancer tumour status. Then, we constructed a ceRNA network of patients with breast cancer based on lncRNA‐miRNA‐mRNA interactions to identify potential prognostic lncRNA biomarkers and understand the regulatory mechanisms at a deep level. From this analysis, we conclude that AL117190.1, COL4A2‐AS1, LINC00184, MEG3 and MIR22HG act as prognostic biomarkers, whose low expression revealed that patients with breast cancer have better overall survival.

In cancers, lncRNAs appear as a prominent layer of previously unrecognized transcriptional regulation, acting simultaneously as an oncogene and tumour suppressor.^27^ The increasing experimental evidence supports that lncRNAs have crucial roles in numerous biological processes, such as epigenetic regulation, DNA damage, cell cycle regulation and participation in signal transduction.^28^ For example, overexpressed lncRNA HOTAIR was found to participate in the progression of malignant breast cancer,^29^ colon cancer,^30^ liver cancer^31^ and gastrointestinal stromal tumour.^32^ In addition, dozens of lncRNAs show potential as prognostic markers and therapeutic targets across multiple classes of tumours.^33^ Also, the activity of ceRNA is closely associated with the development of cancers,^34^, ^35^ and in the ceRNA network, lncRNAs could competitively bind miRNAs to regulate target mRNA expression.^36^

Maternally expressed gene 3 (MEG3), also known as GTL2, FP504 or LINC00023, is an imprinted gene with maternal expression, located in human chromosome 14q32.^37^, ^38^ It was reported that MEG3 plays a key role in regulating many functions in cell growth and development via different mechanisms.^39^ Increasing evidence showed that, in addition to inhibition of cancer cell growth, MEG3 could also play a vital role in stimulation of p53‐mediated transcriptional activation and selective activation of p53 target genes.^40^, ^41^ In relevant research of gastric cancer, Sun et al drew an important conclusion that the low‐expression level of MEG3 was more common in tumour tissues rather than in adjacent healthy tissues; also, the reduction of MEG3 expression level in gastric cancer is related with not only poor survival but also promotion of cell proliferation.^42^ Additionally, low MEG3 expression is not only found in gastric cancers, but other cancers as well, because MEG3 acts as a tumour suppressor gene. In breast cancer, reduced expression of MEG3 was correlated with disease‐specific survival of breast cancer.^43^ Our results indicated that expression of MEG3 was low in breast cancer when compared with normal tissues, and low MEG3 expression represented a better overall survival.

Human miR‐22 host gene (MIR22HG) was identified and annotated by The Encyclopedia of DNA Elements (ENCODE) project. MIR22HG was first reported by Torimura et al^44^ and is commonly down‐regulated in tumour tissues and participates in the inhibition of cell proliferation. Abnormal expression of MIR22HG is associated with a variety of tumours. In patients with lung cancer, MIR22HG functions as a cancer suppressor gene and was associated with poor patient survival.^45^ In addition, low expression of MIR22HG was correlated with hepatocellular carcinoma progression and showed potential as a novel prognostic biomarker and treatment target.^46^ A relevant study has shown that as a novel biomarker of thyroid cancer, the expression level of MIR22HG is associated with overall survival and prognosis. From these previous studies, we can consider that MIR22HG is closely related to the prognosis of the tumour. Drawn from our study, we found that low‐expression level of MIR22HR was related to better overall survival of patients with breast cancer. Thus, MIR22HR could be identified as a prognostic independent predictor. However, in terms of its expression and specific function in breast cancer, little is known at present.

According to the consequences of KEGG enrichment pathways, we could reveal the activation role of lncRNAs from the ceRNA network on key pathways that regulate breast cancer development and progression. They were enriched in biological processes closely correlated with breast cancer, such as MAPK signalling pathway, PI3K‐AKT signalling pathway and mTOR signalling pathway. Previous studies have demonstrated that mutations in PI3K‐AKT and mTOR pathway are related with cell transformation, tumour occurrence and progression.^47^, ^48^, ^49^ Based on KEGG pathways, we could dive into more details of these lncRNAs from the ceRNA network in the future.

In our work, we applied an integrated bioinformatics approach, WGCNA, to establish the lncRNA‐miRNA‐mRNA ceRNA network for breast cancer. Notably, beginning with TCGA RNA transcript profiles collected from breast cancer specimens, via WGCNA and ceRNA bioinformatics analysis, we identified six lncRNAs, of which three (AL117190.1, COL4A2‐AS1 and LINC00184) are unknown. Most importantly, low expression of AL117190.1, COL4A2‐AS1 and LINC00184 were correlated with better overall survival and the diagnostic and prognostic values need to be further clarified by independent validation. Nevertheless, the verification steps of the experiments use to generate the data utilized in this study lacked the involvement of these lncRNAs, which was a limitation in our study.

In summary, we have identified five novel lncRNAs (AL117190.1, COL4A2‐AS1, LINC00184, MEG3 and MIR22HG) related to prognosis of breast cancer, which could act as underlying prognosis biomarkers for breast cancer. Our study has contributed to further understanding the molecular mechanism of breast cancer and provided novel insights into the use of breast cancer drugs and prognosis.

## CONFLICT OF INTEREST

The authors confirm that there are no conflicts of interest.

## AUTHORS CONTRIBUTION

Changgang Sun, Tingting Zhang and Yan Yao were involved in the concept and design of the study. Tingting Zhang and Yan Yao drafted the manuscript. All authors participated in acquisition, analysis and interpretation of the data; revised the manuscript; and read and approved the final version.

## Data Availability

Data comes from TCGA database, which is a public open platform.
